# Propolis attenuates diabetes-induced testicular injury by protecting against DNA damage and suppressing cellular stress

**DOI:** 10.3389/fphar.2024.1416238

**Published:** 2024-07-11

**Authors:** Ahmed M. Ashour

**Affiliations:** Pharmacology and Toxicology Department, College of Pharmacy, Umm Al-Qura University, Makkah, Saudi Arabia

**Keywords:** propolis, streptozotocin, nicotinamide, PCNA, Bcl-2, rats

## Abstract

**Introduction:** Propolis has a wide range of biological and pharmacological actions, including antioxidant properties—particularly its phenolic and flavonoid constituents—that could potentially protect the reproductive system from oxidative damage.

**Method:** Four groups were allocated 40 male Wistar rats each. The vehicle was given to the first group’s normal control rats negative control. The second, third, and fourth groups of diabetic rats were given vehicle (diabetic control) and propolis orally at 50 and 100 mg/kg, respectively, for 8 weeks. Diabetes was induced in rats via injection of nicotinamide and streptozotocin (STZ). Fasting blood glucose (FBG) and insulin levels, homeostatic model assessment for insulin resistance (HOMA-IR), and semen analysis were assessed. In addition, assessments of serum reproductive hormones, including total testosterone (TTST), estradiol (E2), follicle-stimulating hormone luteinizing hormone (LH), and prolactin (PRL), were measured at the end of the study. Tissue total testosterone, E2, and dihydrotestosterone were also evaluated. Serum and tissue oxidative enzymes, including catalase (CAT), superoxide dismutase, and glutathione peroxidase activities, were examined, and malondialdehyde content was determined. The pancreatic and testicular tissues were histopathologically examined, and proliferating cell nuclear antigen (PCNA) and B-cell lymphoma 2 (Bcl-2) in testicular tissue were immunohistochemically analyzed. Testicular tissue was examined for DNA integrity using a comet assay.

**Results:** Compared to the STZ-control group, propolis greatly decreased FBG levels and improved the glycemic status of diabetic rats. In comparison to the STZ-DC group, propolis increased the number of sperm cells and the percent of morphologically normal and viable sperm in male rats, improving their fertility. Propolis also restored the pancreatic islets, protected the testis from oxidative stress, and increased levels of reproductive hormones in the blood, especially testosterone. Moreover, propolis at high doses demonstrated a strong positive response for Bcl-2 and a negative expression of proliferating cell nuclear antigen in spermatogenic cells.

**Conclusion:** The data obtained strongly indicate that STZ causes severe impairments to the testis whereas propolis, acting as an antioxidant, protects against the adverse effects of STZ on the testis.

## 1 Introduction

Diabetes mellitus (DM) is an important health issue for contemporary nations, with an upsurge in its prevalence. DM describes a metabolic disorder with several causes; it is defined by persistent high blood sugar levels and disturbances in the metabolism of carbohydrates, fats, and proteins. These arise from deficiencies in the secretion of insulin, its action, or both (Ding et al., 2015).

Type 2 DM (T2DM) is distinguished by insulin resistance, which might be accompanied by a substantially diminished capacity for insulin secretion. It is well-established that lifestyle factors such as obesity, stress, lack of physical activity, and a deficient diet play a significant role in the development of T2DM. Long-term damage, dysfunction, and failures of multiple organs, such as retinopathy with possible blindness, nephropathy resulting in renal failure, peripheral neuropathy with foot ulcer risk, amputation risk, and autonomic neuropathy causing gastrointestinal, genitourinary, cardiovascular, and sexual dysfunction could be caused by DM. It has also been shown that glucose metabolism is crucial for sperm cells. DM, either type 1 or type 2, has been shown to have a negative impact on male fertility, particularly on sperm quality factors like motility, DNA integrity, and seminal plasma composition. Moreover, diabetes negatively impacts sexual functions, including chromatin quality, nuclear DNA fragmentation, and semen characteristics. This has been demonstrated by several investigations on both humans and animals (Kilarkaje et al., 2014; Ding et al., 2015).

DM impacts various cellular mechanisms and pathways with significant implications for male reproductive function. It significantly alters the endocrine regulation of spermatogenesis, which can lead to impaired sperm quality and/or impaired functionality in diabetic male individuals (Alves et al., 2013).

During their passage through the epididymis, spermatozoa develop forward motility and the capability to fertilize. The ability of mammalian spermatozoa to produce reactive oxygen species (ROS), including hydrogen peroxide (H_2_O_2_) and the superoxide anion (O^−^_2_), is closely linked to the basic storage and maturation processes of the epididymis. The production of these extremely reactive metabolites plays a crucial role in the signal transduction pathways that regulate sperm capacitation (Aitken, 2002).

Male infertility has been linked to sperm dysfunction, primarily as a result of damage caused by ROS—specifically H_2_O_2_ and (O^−^_2_). The high quantity of unsaturated fatty acids in spermatozoa makes them susceptible to oxidative stress, which can then initiate a lipid peroxidation chain reaction. This is a major factor in the etiology of male infertility because it causes the spermatozoa to lose their function and results in the decreased fluidity of the sperm plasma membrane (Sharma and Agarwal, 1996; Capucho et al., 2012).

Propolis; or bee glue, is a naturally occurring beekeeping product, with the quantity of each ingredient varying based on its source planet. It has a wide range of biological and pharmacological actions, including analgesic, tissue-regenerative, antibacterial, antioxidant, and anti-inflammatory effects (Seven et al., 2020). Furthermore, it has been shown that testicular tissue oxidative damage negatively impacts the reproductive system and that, therefore, antioxidants may be useful in avoiding or lessening this damage. Consequently, it has been documented that the antioxidant properties of propolis, particularly its phenolic and flavonoid constituents, protect the reproductive system from oxidative damage (Öğretmen et al., 2014).

The purpose of this study was to investigate how propolis affects male fertility in rats with diabetes. This information may help clarify how propolis acts on this reproductive organ, guaranteeing its favorable effects and maybe aiding in the development of successful treatments for male infertility in diabetics.

## 2 Materials and methods

### 2.1 Chemicals

Propolis was provided by Puritan’s Pride, United States. Streptozotocin (STZ) was acquired from Sigma-Aldrich, United States. Nicotinamide was procured from Merck, France. For other additional chemicals included in the experiment, the highest analytical grade available was selected.

### 2.2 Animals

A total of 40 mature male Wistar rats weighing 180–200 g were obtained from Umm Al-Qura University’s animal house colony, Makkah, Saudi Arabia. The animals were housed in standard cages that were pathogen-free, kept at a steady room temperature, and had regular cycles of light and dark. They were given unrestricted access to food and water. The experiment started after the rats had a week to adjust to their new surroundings. The study was carried out in compliance with the guidelines of Umm Al-Qura University’s Standing Committee of Bioethics Research, which complies with the National Regulations on Animal Welfare, authorization number HAPO-02-K-012-2024-02-2042.

### 2.3 Induction of diabetes

In overnight fasted rats, two consecutive intraperitoneal injections of nicotinamide (NA) and streptozotocin (STZ) were done to induce diabetes (Abdel-Rahman et al., 2020a). The rats were given an intraperitoneal injection of a recently prepared STZ solution (45 mg/kg) in 0.1 M citrate buffer (pH 4.5) 15 minutes after the administration of NA (110 mg/kg) dissolved in sterile saline (Yusufoglu et al., 2015). All rats received injections of NA-STZ except for those in the negative control group, which instead received the vehicle (distilled water, DW). The rats were allowed free access to a 10% (w/v) glucose solution for 24 h after receiving a 6-h NA-STZ injection. Following a 48-h NA-STZ injection, the fasting blood glucose (FBG) level was measured in accordance with Trinder (1969). Rats classified as diabetics and assigned to additional screening and testing were those with FBG levels greater than 200 mg/dL (Yusufoglu et al., 2015).

Male rats with diabetes were fed a high-fat diet (HFD) of 18% protein, 41% carbohydrates, and 41% fat (as a percentage of total kcal). A standard diet was given to the negative control rats (3% fat, 48.8% carbohydrate, and 21% protein).

### 2.4 Experimental design

**FIGURE 1:**
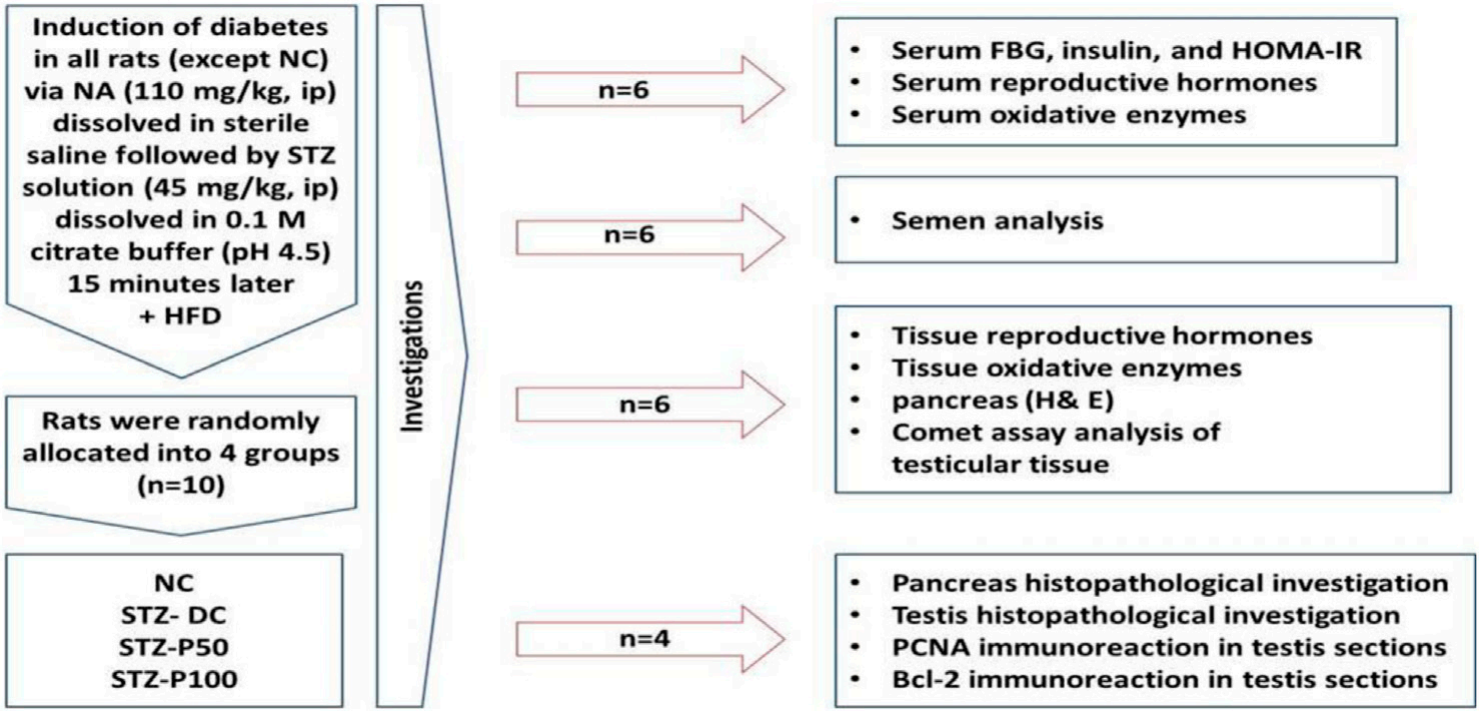
Schematic representation of the experimental design.

Ten diabetic rats were randomly assigned to groups (Figure 1). Throughout the 8-week experiment, the first and second groups—designated as the “diabetic” and “negative control” (NC) groups, respectively—received nothing but the vehicle of distilled water (DW) orally. The third and fourth groups received oral doses of 50 and 100 mg/kg per day, respectively, of propolis dissolved in DW (STZ-P50 and STZ-P100) (Abdel-Rahman et al., 2020b).

### 2.5 Euthanasia, blood and tissue samples

At the termination of the study, anesthesia with ketamine (70 mg/BW) and xylazine (10 mg/BW) was administered to the experimental animals. Anesthetized rats were decapitated after blood samples were drawn from the tail vein, and their vas deferens and testicles were promptly dissected out (Saeedan et al., 2023).

### 2.6 Measurement of FBG, insulin, and HOMA-IR

The levels of insulin and fasting blood glucose (FBG) were measured 8 weeks after medication started. Blood was drawn into sampling tubes from the tail vein while under light anesthetic. Serum was then separated to determine insulin levels by centrifuging blood samples at 3,500 rpm for 15 min (Anderson et al., 1992). HOMA-IR was then expressed as [fasting glucose (mg/dL) × 0.0555 × fasting serum insulin (mUI/L)/22.5] (Matthews et al., 1985).

### 2.7 Seminal parameters

The scrotum underwent a horizontal incision. A specimen of semen was taken from the cauda epididymis. The semen samples were examined as soon as they were collected. As per Khalaji et al. (2018), sperm count, motility, morphology, and viability were measured. Using Tyrode’s buffer solution, mature spermatozoa were extracted from the cauda of the right epididymis after fine mincing, with a final volume of 3.0 mL at 37°C. To measure motility and sperm cell count, a sample of this sperm suspension was instantly put on a heated hemocytometer, and each sperm was counted in 20 fields (×40). A 1.0 µL piece of the sperm suspension was incubated with eosin-nigrosin, and sperm morphology was assessed by classifying the sperm as normal or abnormal based on a fine smear inspected under a ×1000 light microscope (two heads or two tails, short head, no hook, excessive hook, amorphous, and pin head).

### 2.8 Measurement of serum and testicular tissue hormones

Using an enzyme-linked immunoassay kit (Biogen^®^, Saudi Arabia), serum reproductive hormone levels for total testosterone (TTST), estradiol (E2), follicle-stimulating hormone (FSH), luteinizing hormone (LH), and prolactin (PRL) were measured.

### 2.9 Measurement of serum and testicular tissue antioxidants

Oxidative enzymes including catalase (CAT) activity were determined using the ammonium molybdate method; superoxide dismutase (SOD) activity was determined using the hydroxylamine method; glutathione peroxidase (GPx) activity was determined using the dithio-nitrobenzoic acid method; malondialdehyde (MDA) content was determined using the TBA method (Marí et al., 2010; Masella et al., 2005; Niki, 2009; Jin et al., 2000).

### 2.10 Histopathological examination of pancreatic and testicular tissues

Pancreas and testis specimens were preserved in a 10% neutral buffer formalin solution, then cut, rinsed with water, dried in ascending concentrations of ethyl alcohol, and then washed in xylene and embedded in paraffin. Hematoxylin and eosin stain was applied to thin slices (4-6µ) after processing (Bancroft and Gamble, 2007). The fibrosis score in the pancreatic sections was performed according to Lu et al. (2007). Spermatogenesis scoring was done according to Johnsen (1970) as modified by Holstein et al. (2003).

### 2.11 Immunohistochemical analysis of proliferating cell nuclear antigen (PCNA) and B-cell lymphoma 2 (Bcl-2) in testicular tissue

As per Albaqami et al. (2024), paraffin slices of testicles were adhered to positively charged slides via the avidin–biotin–peroxidase complex (ABC) technique, primary antibody. After these antibodies were incubated on sections from each group, the chemicals needed for the ABC technique were added (Vectastain ABC-HRP kit, Vector laboratories). To identify the antigen–antibody complex, diaminobenzidine (DAB, manufactured by Sigma) was used to color the marker expression and label it with peroxidase. The primary or secondary antibodies were swapped out for non-immune serum to create negative controls. Sections stained with IHC were inspected under an Olympus microscope (BX-53). PCNA and Bcl-2 rabbit polyclonal antibodies were obtained from Wuhan Servicebio Technology Co. Ltd., China (Cat. no. GB11010, GB114830). Immunohistochemistry results were scored by calculating the reaction area percent in ten microscopic fields using ImageJ 1.53t, Wayne Rasband and contributors, National Institutes of Health, United States.

### 2.12 Evaluation of DNA integrity using comet assay

The integrity of sperm DNA in testis tissue samples was assessed by alkaline single gel electrophoresis (comet assay), with minor changes, as per Frenzilli et al. (2004). Tissue suspension was prepared by mincing 0.1 g testes tissue using scissors in Ca++ and Mg++ free phosphate-buffered saline (PBS) containing 20 mM EDTA. Samples were then lysed overnight at 4°C in a vertical coplin jar containing lysis buffer (2.5 M NaCl, 1 mM EDTA, 10 mM TRIS, pH 10, 1% N-lauroylsarcosine, 1% triton X-100% and 10% DMSO). After being washed once in distilled water, the slides were covered with freshly prepared alkaline electrophoresis buffer (300 mM NaOH, 1 mM Na2-EDTA, pH 13) for unwinding for 20 min. They were then immersed in the alkaline electrophoresis solution and electrophoresed for 30 min at 20 V/<300 mA at 4°C. Directly after that, slide neutralization was performed thrice by (0.4 M Tris HCL, pH7.5), 5 min each time, followed by DNA precipitation using absolute ethanol for 10 min. The slides were then kept in a wetted dark box until examination. Slide staining and visualization were done by staining each gel with 50 µL of 20 μg/mL ethidium bromide. The slides were visualized using ×40 objective of Leica epifluorescent microscope (Green filter: N2.1 with Excitation filter: BP 515-560, Dichromatic Mirror: 580, Suppression filter: LP 590). The images for the cell nuclei were digitalized with truechrome retina screen camera version 4.2 build 5001 (copyright Tucsen Photonics Co. ltd.). A minimum 50 cell nuclei for each sample were measured using image analysis software TriTek CometScore™ freeware v1.5. to obtain the percentage of DNA in the head, tail, tail moment, and olive moment.

### 2.13 Statistical analysis

Before proceeding with statistical analysis with Graph Prism^®^ (version 9, United States), data values were checked for normality using the Shapiro test and for heteroscedasticity with the Brown–Forsythe test. All the data obtained were presented as mean ± SEM. Statistical analysis was done using one-way analysis of variance (one-way ANOVA) followed by Tukey’s test to determine intergroup variability. A probability level of less than 0.05 was accepted as statistically significant. For non-parametric data, values were presented as median ± interquartile range and analyzed by the Kruskal–Wallis test followed by Dunn’s test. The significance level was set as *p* ≤ 0.05 for all statistical tests.

## 3 Results

### 3.1 Effect on blood glucose, insulin, and HOMA-IR levels

Table 1 depicts the levels of fasting blood glucose (FBG), insulin, and HOMA-IR of male diabetic rats after 8 weeks of medication. Propolis (50 and 100 mg/kg) significantly decreased FBG levels compared to the streptozotocin-diabetic control (STZ-DC) group by 55.2% and 56.6%, respectively. The insulin levels of diabetic rats treated with propolis (50 and 100 mg/kg) were significantly improved compared to STZ-DC rats (*p* ≤ 0.05) by 38.1% and 64.8%, respectively. HOMA-IR was significantly stabilized in propolis-treated rats compared to the STZ-DC group by 52.2% and 58.6%, respectively.

**TABLE 1:** Effect of propolis on serum levels of FBG, insulin, and HOMA-IR in STZ-diabetic rats.

Group	FBG (mg/dL)	Insulin (uIU/mL)	HOMA-IR
NC	91.9^b^ ±1.61	4.01^b^ ±0.19	0.95^b^ ±0.03
STZ-DC	312.2^a^ ±8.40	1.76^a^ ±0.09	2.49^a^ ±0.08
STZ-P50	140.0^ab^ ± 5.98	2.43^ab^ ± 0.18	1.19^b^ ±0.10
STZ-P100	135.2^ab^ ± 9.64	2.90^ab^ ± 0.14	1.03^b^ ±0.08

Data presented as mean ± SEM (n = 6).

^a^ Statistically significant from the STZ-DC group at *p* ≤ 0.05.

^b^ Statistically significant from the NC group at *p* ≤ 0.05.

Multiple group comparisons made by analysis of variance (ANOVA) and Tukey’s multiple comparison *post hoc* test.

NC, negative control; STZ-DC, streptozotocin- diabetic control; STZ-P50, propolis 50 mg/kg; STZ-P100, propolis 100 mg/kg.

FBG, fasting blood glucose; HOMA-IR, homeostatic model assessment for insulin resistance.

### 3.2 Effect on seminal parameters

As shown in Table 2, the sperm characteristics of male rats were recorded. STZ-DC rats revealed a significantly greater decline in sperm motility (53.5% ± 1.43%) than negative control (NC) rats (73.6% ± 1.27%). An administration of propolis (50 and 100 mg/kg) for 8 weeks markedly improved sperm motility (59.9% ± 0.87% and 70.0% ± 1.34%, respectively) compared to STZ-DC rats. Meanwhile, for STZ-DC rats, sperm count was 35.0 ± 0.45 million/mL, less than the NC count (59.2 ± 1.27 million/mL). Administration of propolis (50 and 100 mg/kg) for 8 weeks resulted in significantly increased sperm cell count (44.7 ± 1.52 and 57.3 ± 0.77 million/mL, respectively) compared to STZ-DC rats.

**TABLE 2:** Effect of propolis on seminal parameters in STZ-diabetic rats.

Group	Seminal parameters			
	Sperm motility (% motile sperm)	Sperm count (x 10^6^ sperm/mL)	Sperm morphology (% normal cells)	Sperm viability (% viable sperm)
NC	73.6^b^ ±1.27	59.2^b^ ±1.27	95.9^b^ ±3.65	72.1^b^ ±1.60
STZ-DC	53.5^a^ ±1.43	35.0^a^ ±0.45	53.2^a^ ±1.59	48.3^a^ ±1.22
STZ-P50	59.9^ab^ ± 0.87	44.7^ab^ ± 1.52	62.9^ab^ ± 1.07	55.4^ab^ ± 0.45
STZ-P100	70.0^b^ ±1.34	57.3^b^ ±0.77	91.7^b^ ±1.90	68.1^b^ ±1.68

Data presented as mean ± SEM (*n* = 6).

^a^ Statistically significant from the NC group at *p* ≤ 0.05.

^b^ Statistically significant from the STZ-DC group at *p* ≤ 0.05.

Multiple group comparisons made by ANOVA and Tukey’s multiple comparison *post hoc* test.

NC, negative control; STZ-DC, streptozotocin- diabetic control; STZ-P50, propolis 50 mg/kg; STZ-P100, propolis 100 mg/kg.

An investigation of semen samples for the morphological structure of sperm (Table 2) showed a significantly lower percentage of morphologically normal spermatozoa in the STZ-DC group (53.2% ± 1.59%) than the NC group (95.9% ± 3.65%). The furthermost types of abnormalities were coiled and bent tails and detached head. In propolis treated groups (50 and 100 mg/kg), the percentage of morphologically normal sperm were 62.9% ± 1.07% and 91.7% ± 1.90%, respectively.

The percentage of viable sperm in STZ-DC rats (48.3% ± 1.22%) was significantly less than in NC rats (72.1% ± 1.60%). The sperm viability in propolis-treated rats (50 and 100 mg/kg) was significantly enhanced (55.4% ± 0.45% and 68.1% ± 1.68% respectively) compared to STZ-DC group.

### 3.3 Effect on serum reproductive hormones

The effects of propolis administration to diabetic rats on serum reproductive hormones are illustrated in Table 3. The STZ-DC group revealed a significant decrease in the serum TTST (10.4 ± 0.49 ng/mL), E_2_ (36.1 ± 0.99 pg/mL), FSH (2.5 ± 0.22 ng/mL), LH (2.1 ± 0.15 ng/mL), and PRL (10.0 ± 0.40 ng/mL) compared to the NC group (15.4 ± 0.55 ng/mL, 44.5 ± 1.23 pg/mL, 5.3 ± 0.28 ng/mL, 3.9 ± 0.15 ng/mL, and 17.6 ± 0.25 ng/mL, respectively). Serum reproductive hormones exhibited a significant increase in rats administered the high dose of propolis (100 mg/kg) compared to STZ-DC rats.

**TABLE 3:** Effect of propolis on serum reproductive hormones in STZ-diabetic rats.

GroupSerum	TTST (ng/mL)	E_2_ (pg/mL)	FSH (ng/mL)	LH (ng/mL)	PRL (ng/mL)
NC	15.4^b^ ±0.55	44.5^b^ ±1.23	5.3^b^±0.28	3.9^b^ ±0.15	17.6^a^ ±0.25
STZ-DC	10.4^a^ ±0.49	36.1^a^ ±0.99	2.5^a^ ±0.22	2.1^a^ ±0.15	10.0^b^ ±0.40
STZ-P50	12.2^a^ ±0.64	37.4 ± 1.34	3.1^a^ ±0.07	2.7^a^ ±0.12	12.4^ab^ ± 0.37
STZ-P100	14.4^b^ ±0.42	42.5^b^ ±1.33	4.6^b^ ±0.16	3.5^b^ ±0.20	15.2^ab^ ± 0.30

Data presented as mean ± SEM (*n* = 6).

^a^ Statistically significant from the NC group at *p* ≤ 0.05.

^b^ Statistically significant from the STZ-DC group at *p* ≤ 0.05.

Multiple group comparisons made by ANOVA and Tukey’s multiple comparison *post hoc* test.

NC, negative control; STZ-DC, streptozotocin- diabetic control; STZ-P50, propolis 50 mg/kg; STZ-P100, propolis 100 mg/kg.

TTST, total testosterone; E2, estradiol; FSH, follicle-stimulating hormone; LH, luteinizing hormone; PRL, prolactin.

### 3.4 Effect on serum oxidative enzymes

**FIGURE 2:**
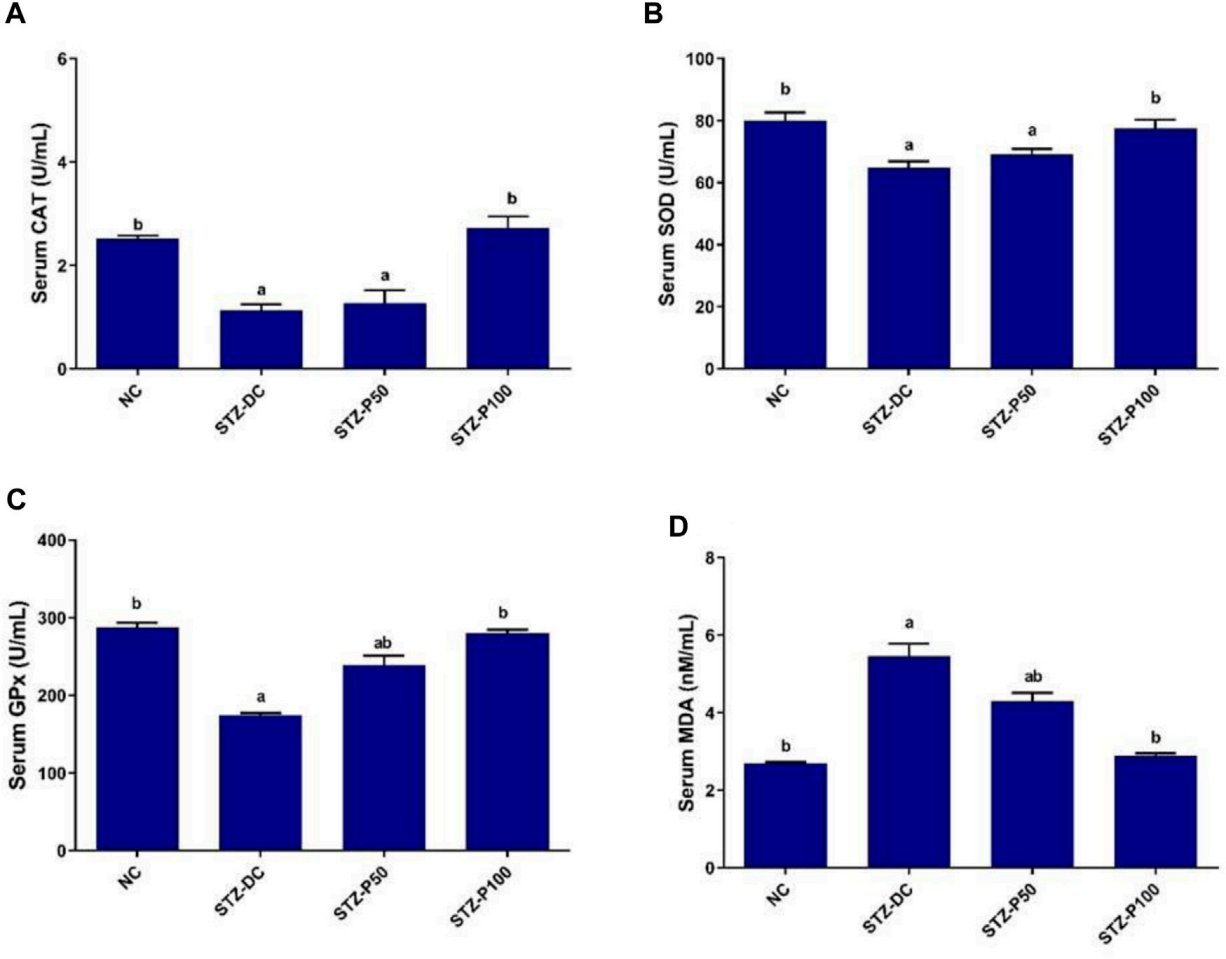
Effect of propolis on serum oxidative enzymes in STZ-diabetic rats. Data presented as mean ± SEM (*n* = 6); ^a^ statistically significant from the NC group at *p* ≤ 0.05; ^b^ statistically significant from the STZ-DC group at *p* ≤ 0.05. Multiple group comparisons made by analysis of variance (ANOVA) and Tukey’s multiple comparison *post hoc* test. CAT, catalase; SOD, superoxide dismutase; GPx, glutathione peroxidase; MDA, malondialdehyde.

The results displayed in Figure 2 reveal that NA-STZ-induced diabetes reduced the activities of the antioxidant enzymes in rat serum compared with the NC rats (*p* ≤ 0.05). The treatment of rats with propolis (100 mg/kg) resulted in significant improvement in the antioxidant profile toward normal levels. Serum CAT, SOD, and GPx activities were significantly increased by 145.5%, 19.8%, and 60.6%, respectively, in the STZ-P100 group compared to STZ-DC rats, whereas serum MDA levels were significantly decreased by 47.3% in the STZ-P100 group compared to STZ-DC rats.

### 3.5 Effect on tissue reproductive hormones

**FIGURE 3:**
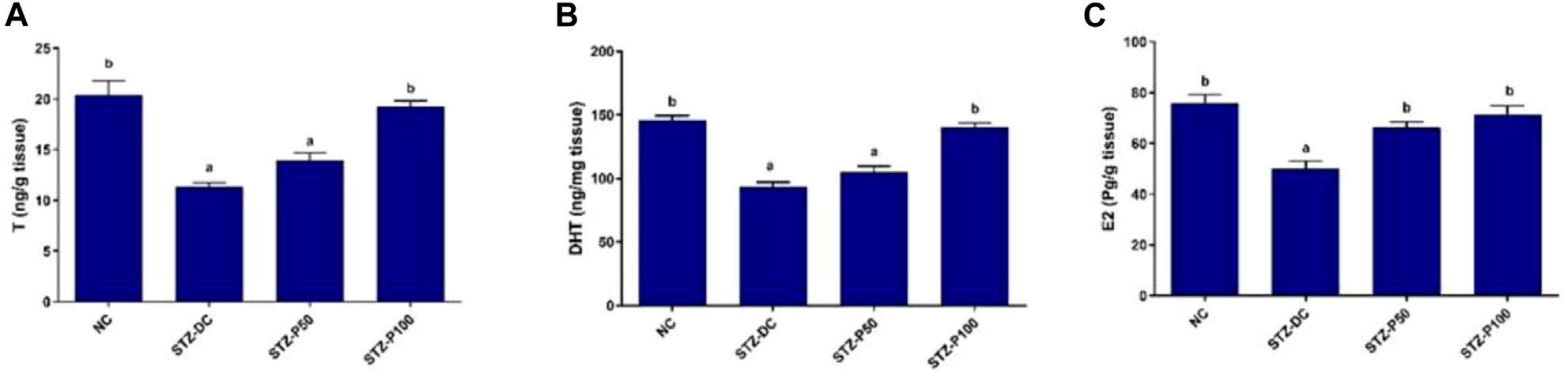
Effect of propolis on tissue reproductive hormones in STZ-diabetic rats. Data presented as mean ± SEM (*n* = 6); ^a^ statistically significant from the NC group at *P*≤0.05; ^b^ statistically significant from the STZ-DC group at *P*≤0.05. Multiple group comparisons made by ANOVA and Tukey’s multiple comparison *post hoc* test. NC, negative control; STZ-DC, streptozotocin- diabetic control; STZ-P50, propolis 50 mg/kg; STZ-P100, propolis 100 mg/kg. TTST, total testosterone; DHT, dihydrotestosterone; E_2_, estradiol.

The effects of propolis treatment on the testicular reproductive hormones of male rats are illustrated in Figure 3. Reproductive hormones (TTST, DHT, and E_2_) in the testicular homogenate of STZ-DC rats significantly decreased by 44.6%, 35.9%, and 33.7%, respectively, compared to NC rats. The STZ-P100 rat group exhibited a significant increase in reproductive hormones levels in testis homogenate compared to STZ-DC rats by 69.9%, 50.4%, and 42.6%, respectively.

### 3.6 Effect on tissue oxidative enzymes

**FIGURE 4:**
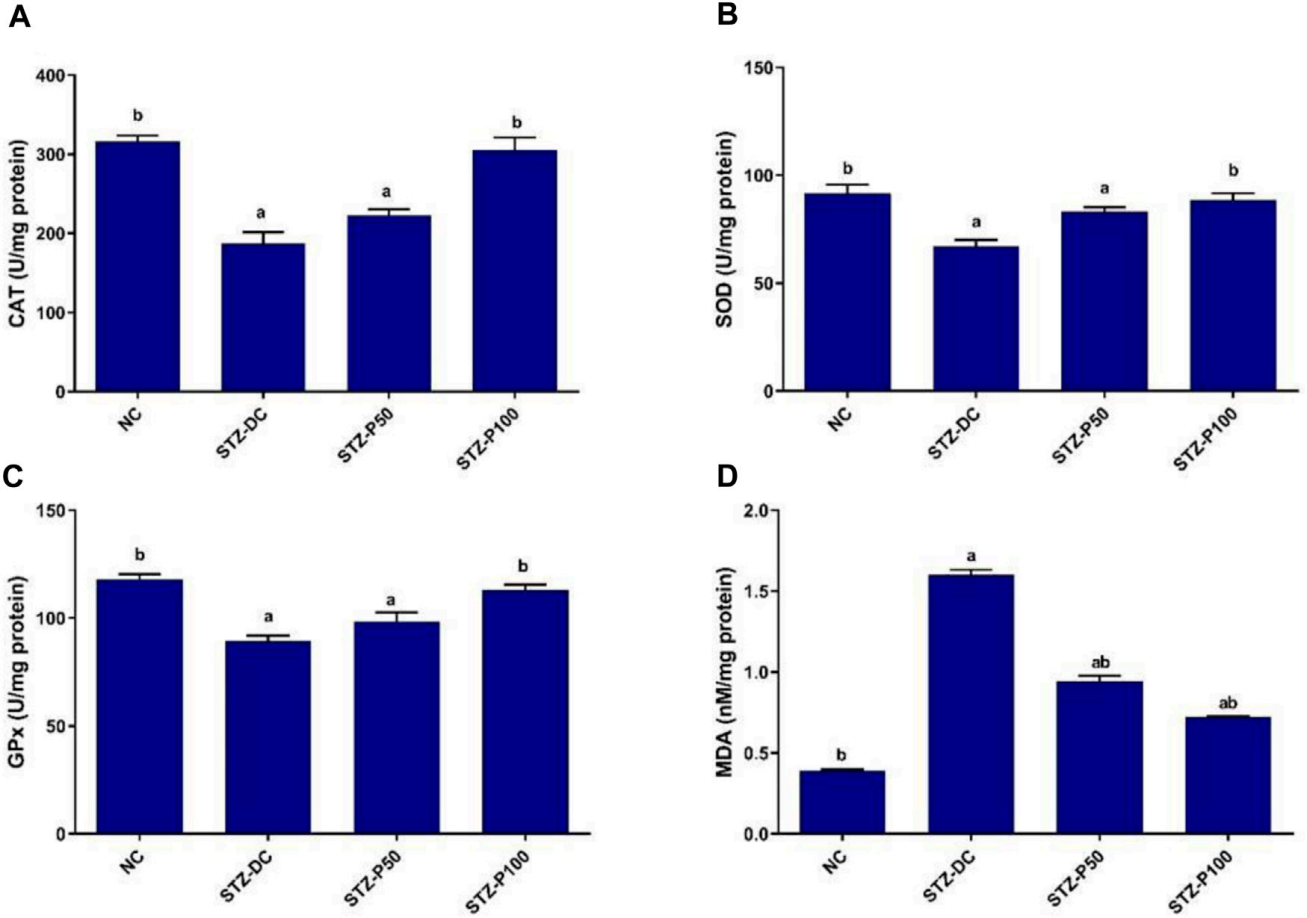
Effect of propolis on tissue oxidative enzymes in STZ-diabetic rats. Data presented as mean ± SEM (n=6); ^a^ statistically significant from the NC group at *P* ≤ 0.05; ^b^ statistically significant from the STZ-DC group at *P* ≤ 0.05. Multiple group comparisons made by ANOVA and Tukey’s multiple comparison *post hoc* test. NC, negative control; STZ-DC, streptozotocin- diabetic control; STZ-P50, propolis 50 mg/kg; STZ-P100, propolis 100 mg/kg. CAT, catalase; SOD, superoxide dismutase; GPx, glutathione peroxidase; MDA, malondialdehyde.

As shown in Figure 4, oxidative enzymes activities in the testicular homogenate of STZ-DC rats revealed significant decrease when compared to those of NC group, while MDA levels showed significant increase in STZ-DC rats compared to NC rats. In contrast, propolis-treated rats (STZ-P100) exhibited significant increase in CAT, SOD, and GPx enzymes activities by 63.0%, 32.1%, and 26.6%, respectively, compared with the STZ-DC group. Additionally, the MDA level in propolis treated rats was significantly decreased by 56.3% compared with STZ-DC rats.

### 3.7 Histopathological investigation of pancreas

**FIGURE 5:**
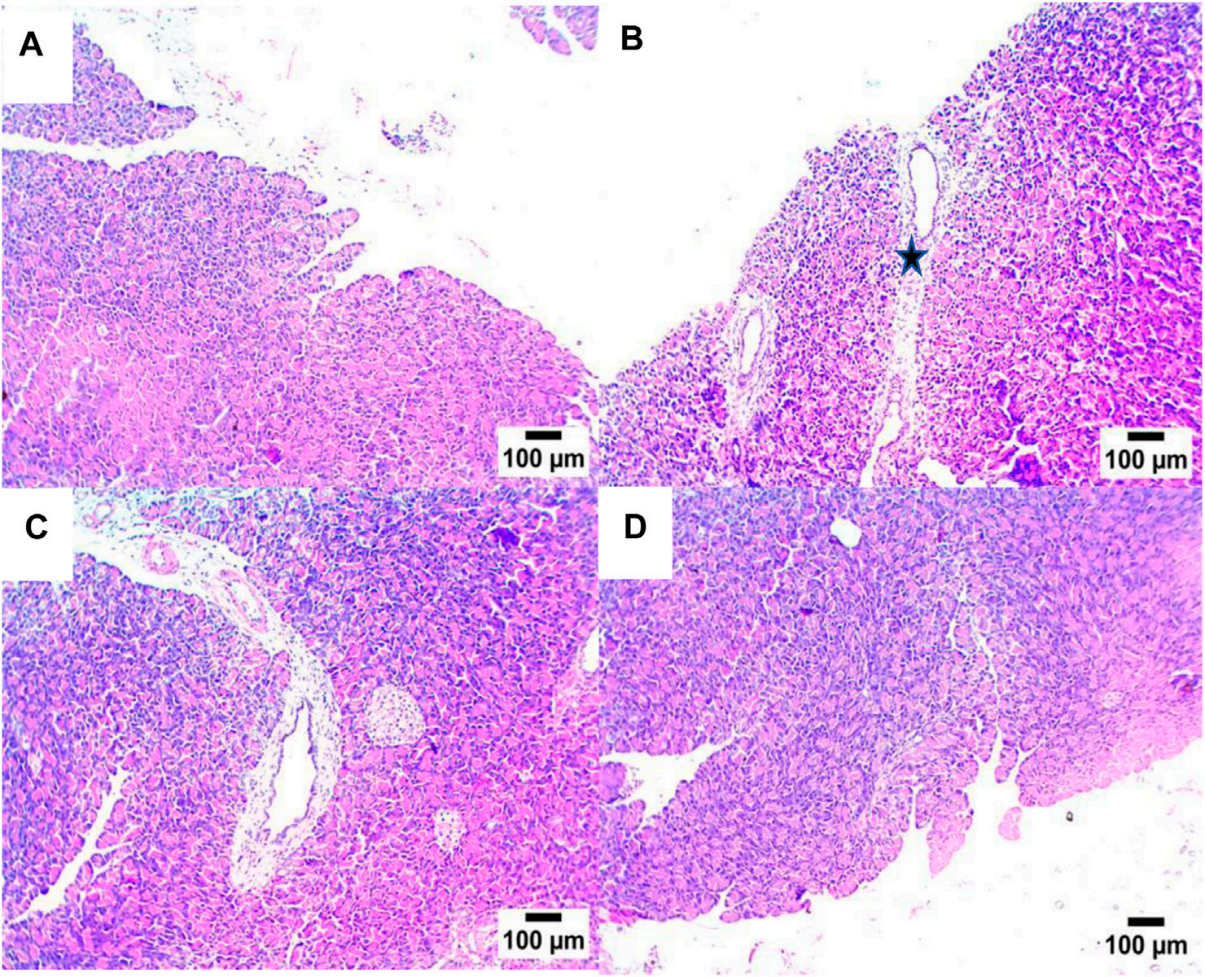
Effect of propolis on pancreas of rats (hematoxylin and eosin stain, H& E, 10X). **(A)** Photomicrograph showing a section of the NC group with a normal histological structure of pancreatic acini; **(B)** section of STZ-DC showing preacinar fibrosis (star); **(C)** section of STZ-P50 showing preacinar fibrosis (star); **(D)** section of STZ-P100 showing mild preacinar fibrosis (arrow) (hematoxylin and eosin stain).

**FIGURE 6:**
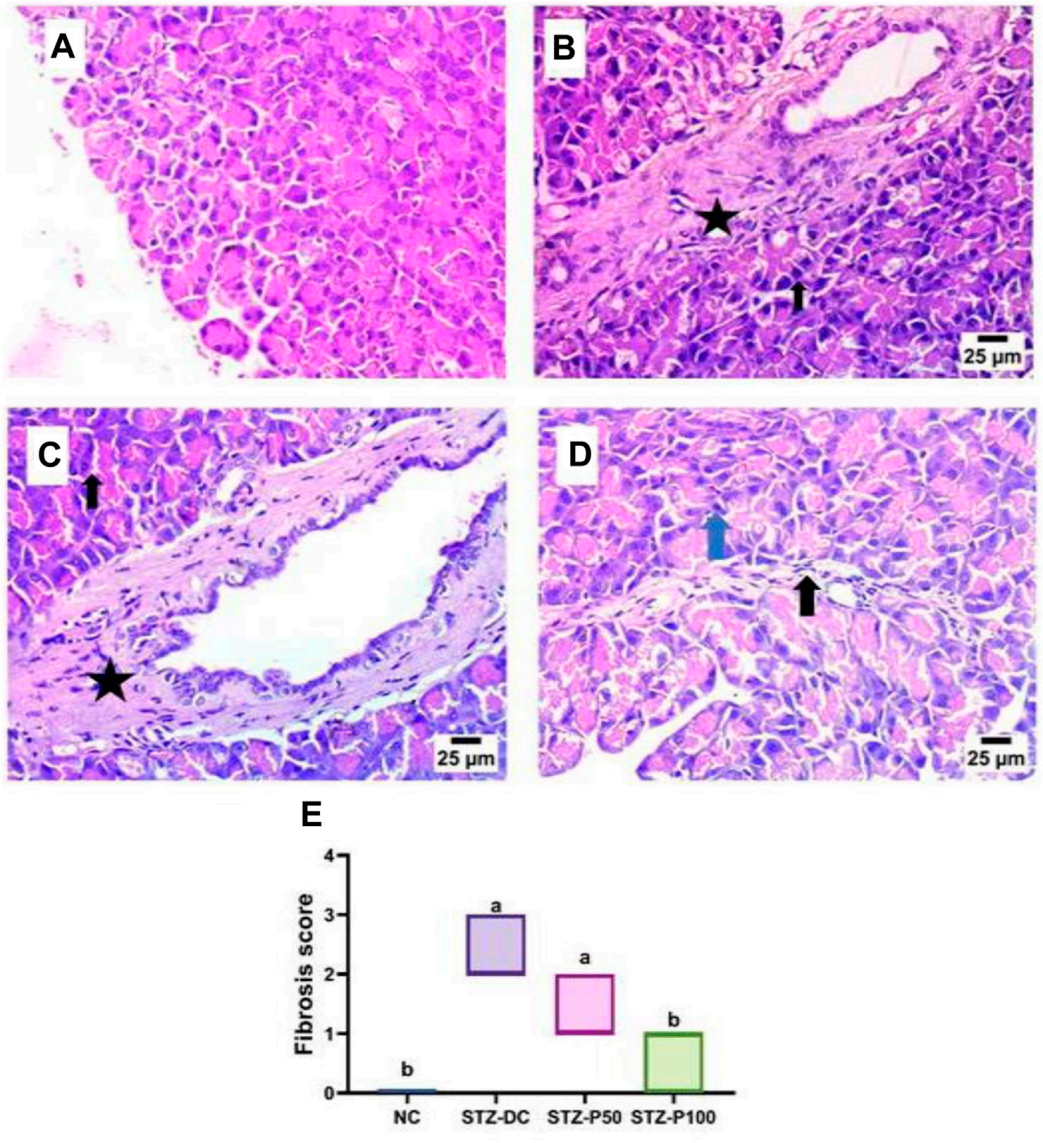
Effect of propolis on the pancreas of rats (hematoxylin and eosin stain, H& E, 40X). **(A)** Photomicrograph of the pancreas section of the NC group showing a normal histological structure of pancreatic acini and beta cells. **(B)** Photomicrograph of the pancreas section of the STZ-DC group showing severe preacinar fibrosis (star) with nuclear pyknosis in pancreatic acini (arrow). **(C)** Photomicrograph of the pancreas section of the STZ-P50 group showing preacinar fibrosis (star) with nuclear pyknosis in some pancreatic acini (arrow). **(D)** Photomicrograph of the pancreas section of the STZ-P100 group showing mild preacinar fibrosis (black arrow) and the presence of pyknotic nuclei in some pancreatic acini (blue arrow). **(E)** Fibrosis score; where ^a^ is statistically significant from the NC and ^b^ is statistically significant from the STZ-DC group at *p* ≤ 0.05).

A histopathological examination of pancreatic tissue of STZ-DC rats showed severe preacinar fibrosis with nuclear pyknosis in pancreatic acini. On the other hand, rats treated with propolis showed mild preacinar fibrosis with pyknotic nuclei in some pancreatic acini (Figures 5, 6).

### 3.8 Histopathological investigation of testis

**FIGURE 7:**
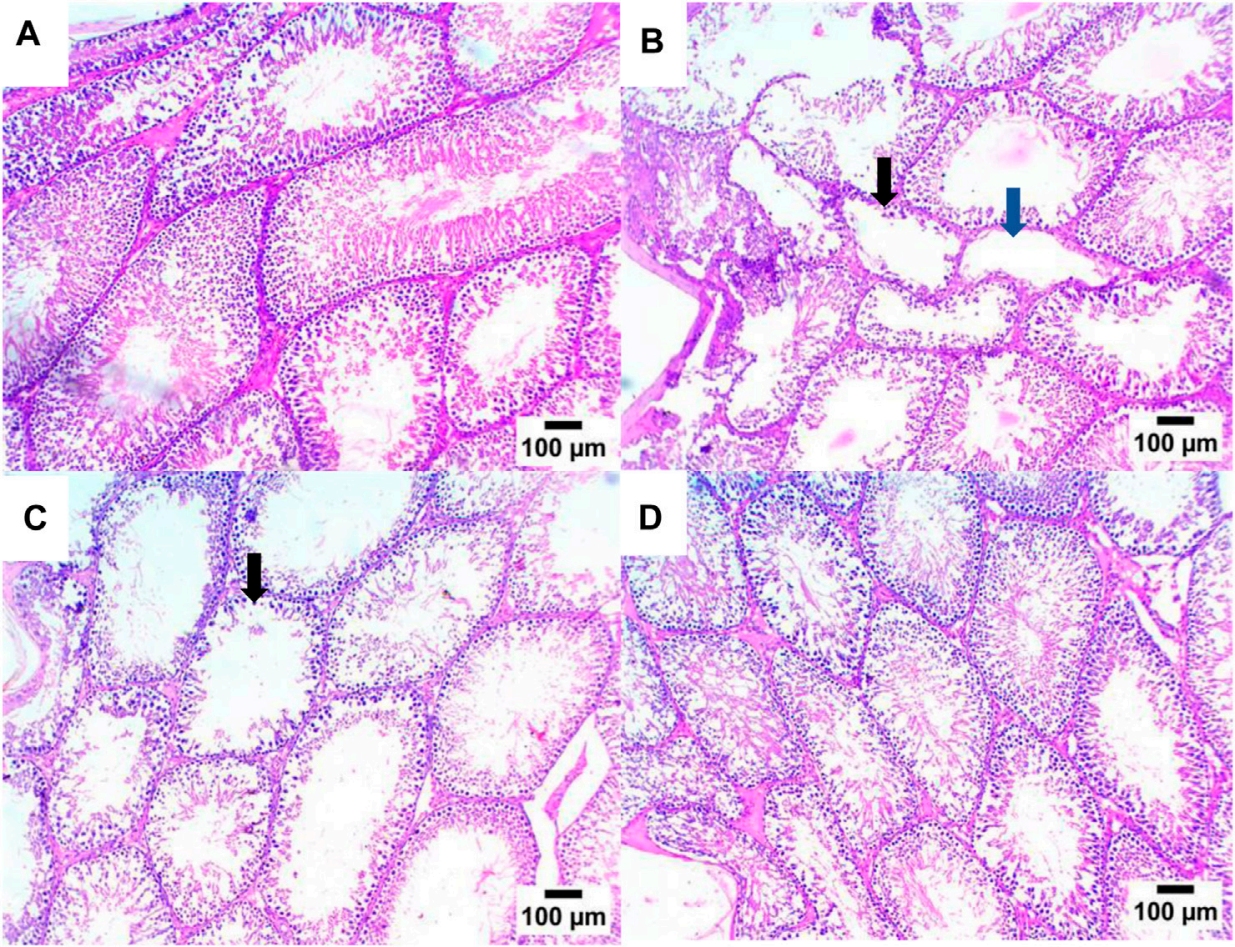
Effect of propolis on the testis of rats (hematoxylin and eosin stain, H& E, 10X). **(A)** Photomicrograph showing a section of the NC group with a normal histological structure of seminiferous tubules; **(B)** section of STZ-DC showing oligospermia in some seminiferous tubules (black arrow) and others showing azospermia (blue arrow); **(C)** section of STZ-P50 showing oligospermia in some seminiferous tubules (arrow); **(D)** section of STZ-P100 showing a normal histological structure of seminiferous tubules.

**FIGURE 8:**
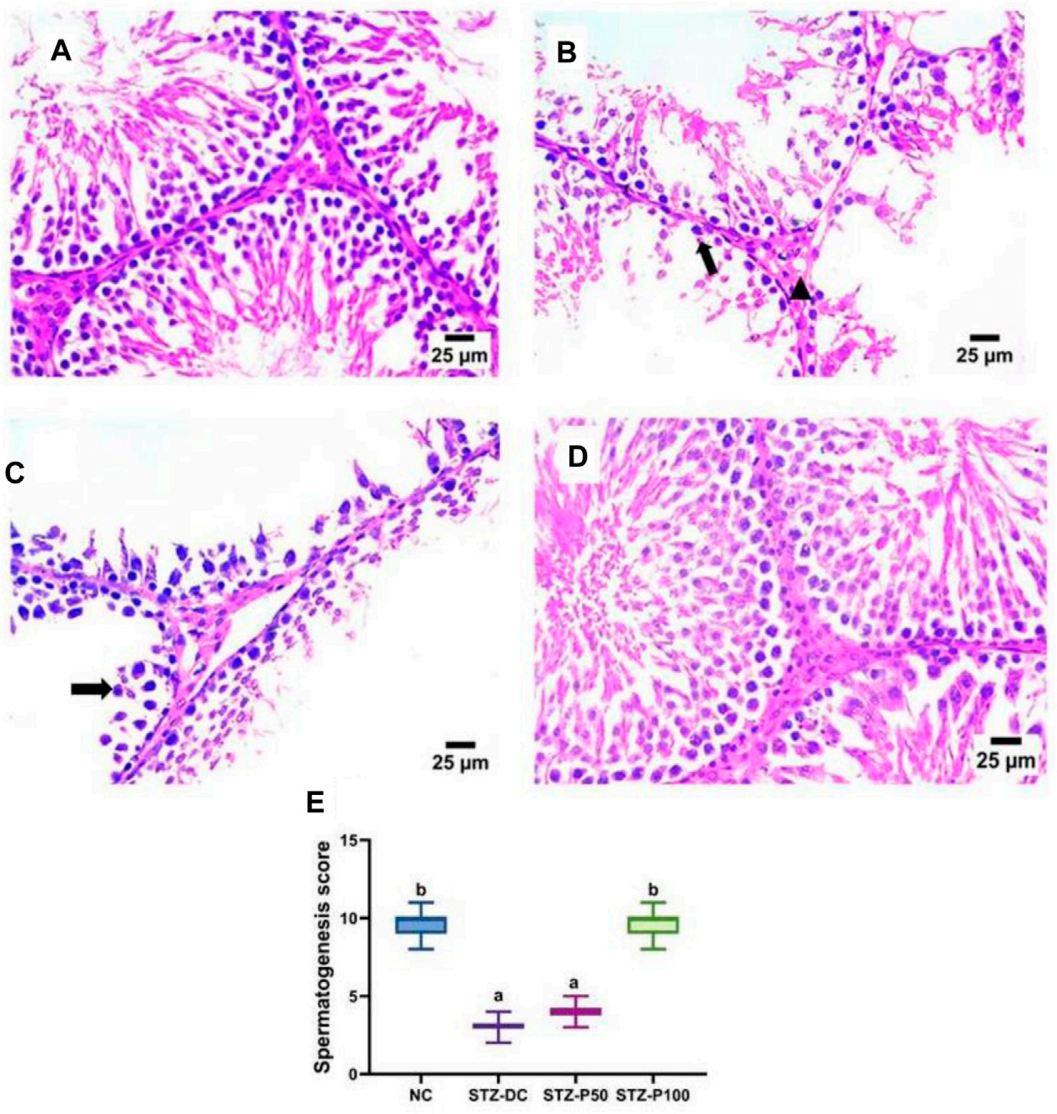
Effect of propolis on the testis of rats (hematoxylin and eosin stain, H& E, 40X). **(A)** Photomicrograph of the testis section of the NC group showing a normal histological structure of seminiferous tubules. **(B)** Photomicrograph of the testis section of the STZ-DC group showing a marked decrease in spermatogenic cells (arrow) with vacuolation in Leydig cells (arrow head). **(C)** Photomicrograph of a section in the testis of the STZ-P50 group showing a decrease in the number of spermatogenic cells (arrow). **(D)** Photomicrograph of a section in the testis of the STZ-P100 group showing a normal histological structure of seminiferous tubules and Leydig cells. **(E)** Spermatogenesis score where ^a^ is statistically significant from NC and ^b^ is statistically significant from the STZ-DC group at *p* ≤ 0.05).

As shown in Figures 7, 8, the histopathologic inspection of the testicular tissue of STZ-DC rats showed a marked decrease in spermatogenic cells with vacuolation in Leydig cells. Propolis-treated rats revealed normal a histological structure of seminiferous tubules and Leydig cells in the high dose group.

### 3.9 Immunohistochemical investigation of testis

**FIGURE 9:**
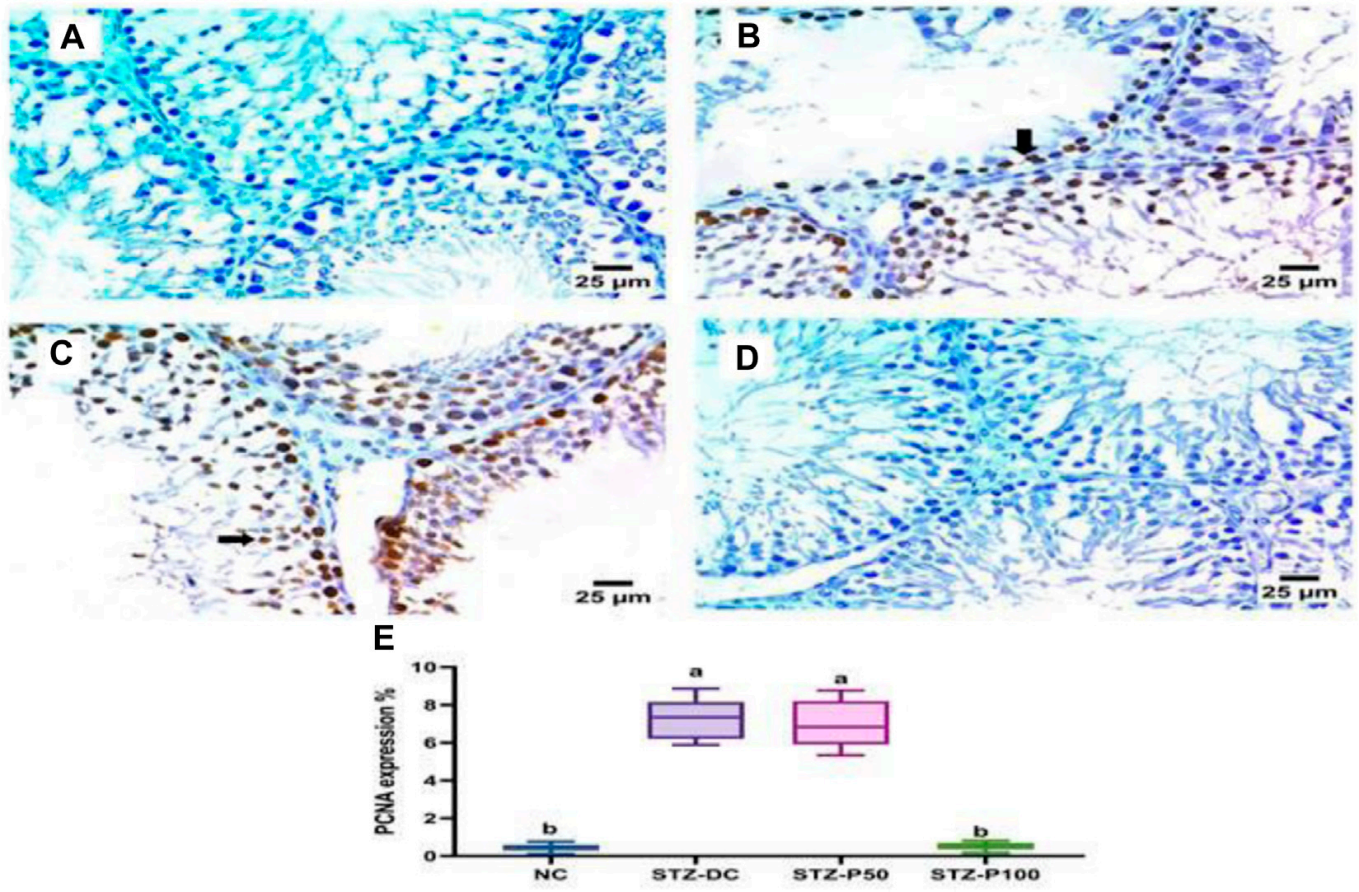
Effect of propolis on PCNA immunoreaction in testis sections (IHC-peroxidase-DAB). **(A)** Photomicrograph of a section in the testis of NC group showing negative expression for PCNA in spermatogenic cells. **(B)** Photomicrograph of a section in the testis of the STZ-DC group showing severe positive expression for PCNA in spermatogenic cells (arrow). **(C)** Photomicrograph of a section in the testis of the STZ-P50 group showing positive expression for PCNA in spermatogenic cells (arrow). **(D)** Photomicrograph of a section in the testis of the STZ-P100 group showing negative expression for PCNA in spermatogenic cells. **(E)** Graph showing the PCNA reaction area percentage, where ^a^ is statistically significant from the NC and ^b^ is statistically significant from the STZ-DC group at *p* ≤ 0.05).

As depicted in Figure 9, the STZ-DC group exhibited severe positive immune reaction against PCNA staining compared to the NC group. Propolis at high dose levels revealed negative expression for PCNA in spermatogenic cells; however, at low dose levels, it failed to decrease PCNA immunoreaction in testis sections. Figure 9E shows the reaction area percentage of PCNA, indicating that the STZ-P100 group significantly varied from the STZ-DC group (*p* ≤ 0.0001).

**FIGURE 10:**
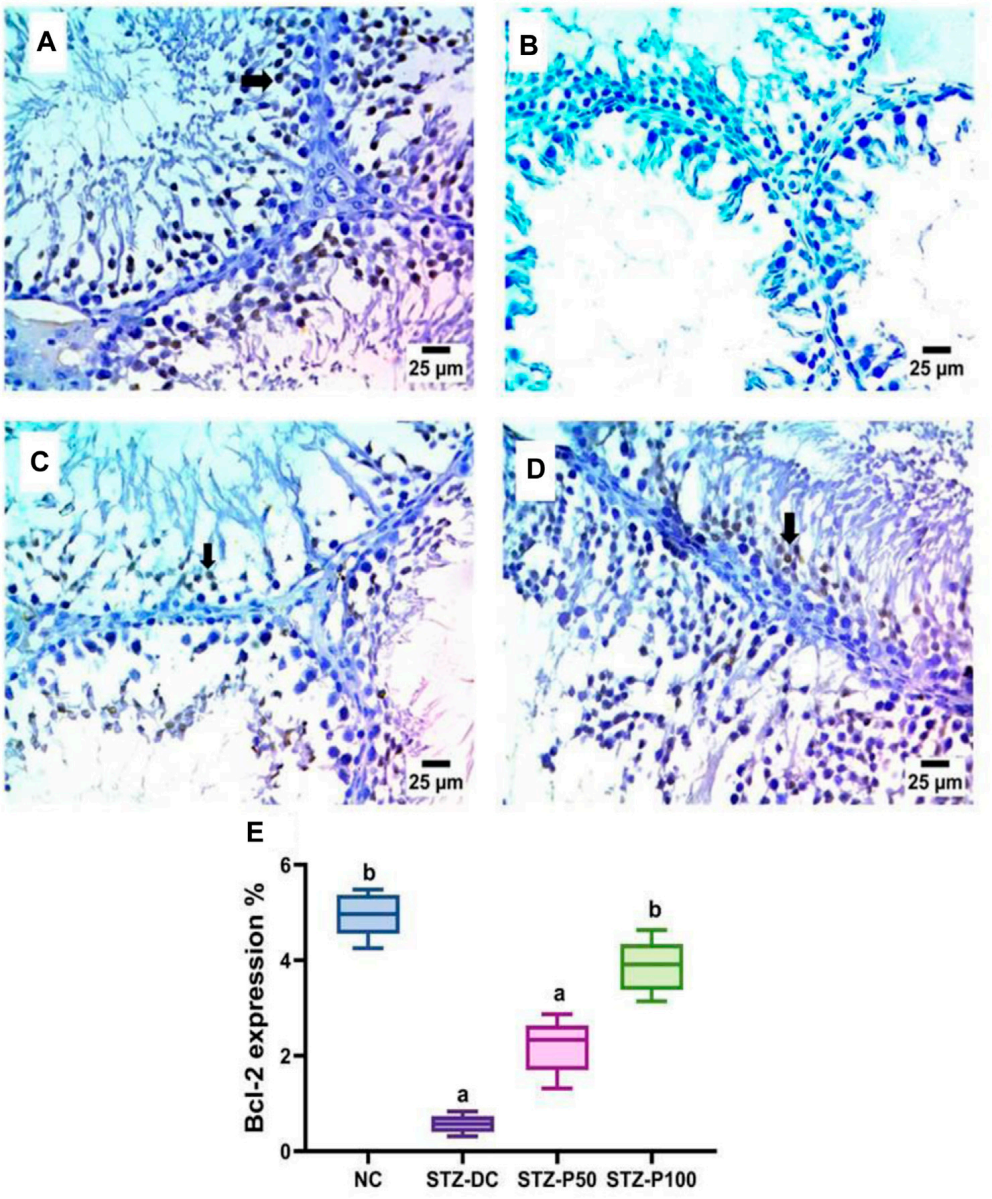
Effect of propolis on Bcl-2 immunoreaction in testis sections (IHC-peroxidase-DAB). **(A)** Photomicrograph of a section in the testis of the NC group showing severe positive expression for Bcl-2 in spermatogenic cells (arrow). **(B)** Photomicrograph of a section in the testis of the STZ-DC group showing negative expression for BCL-2 in spermatogenic cells. **(C)** Photomicrograph of a section in the testis of the STZ-P50 group showing mild positive expression for Bcl-2 in some spermatogenic cells (arrow). **(D)** Photomicrograph of a section in the testis of the STZ-P100 group showing severe positive expression for Bcl-2 in spermatogenic cells (arrow). **(E)** Graph showing the Bcl-2 reaction area percentage where ^a^ is statistically significant from the NC and ^b^ is statistically significant from the STZ-DC group at *p* ≤ 0.05).

Bcl-2 immunoreaction in testis sections is represented in Figure 10. Testicular sections of the STZ-DC group showed negative expression for Bcl-2 in spermatogenic cells. The propolis-treated group (STZ-P100) displayed a severe positive reaction for Bcl-2 in spermatogenic cells. Figure 10E shows the reaction area percentage of Bcl-2 and values, indicating that the means of the STZ-P50 and STZ-P100 groups were significantly increased compared to the STZ-DC group (*p* ≤ 0.0001).

#### 3.9.1 Assessment of DNA fragmentation (COMET)

**FIGURE 11:**
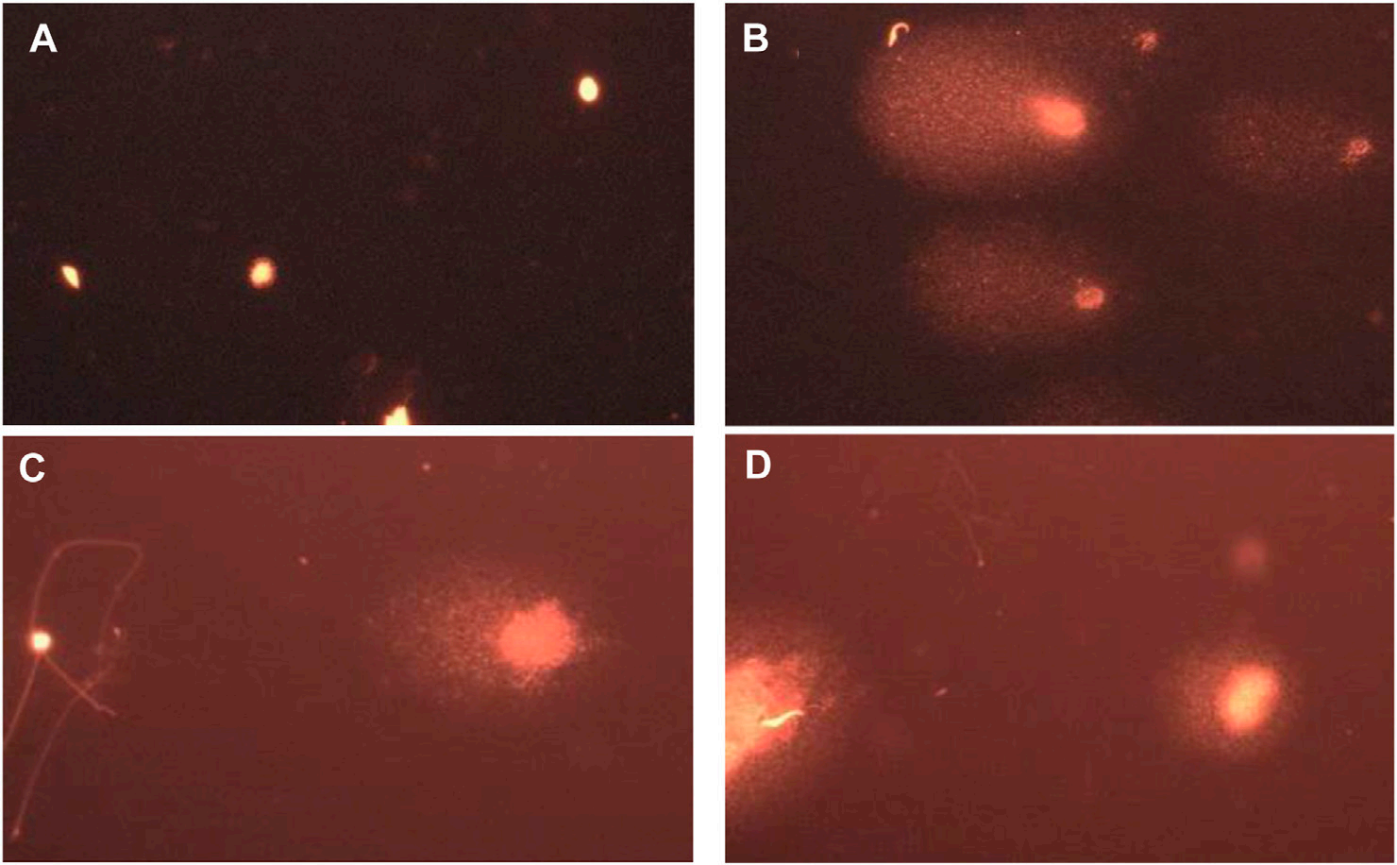
Representative images for comet assay analysis of rat testicular tissue showing **(A)** a nucleoid with intact undamaged DNA (NC), **(B)** damaged DNA (STZ-DC), **(C)** moderate damaged DNA (STZ-P50), and **(D)** mild damaged DNA (STZ-P100).

As shown in Figure 11, photomicrograph B) shows damaged DNA in the diabetic rat group compared to the NC group with intact undamaged DNA A). Rat groups treated with propolis were more protected from DNA damage in testicular samples (photomicrographs C and D).

**FIGURE 12:**
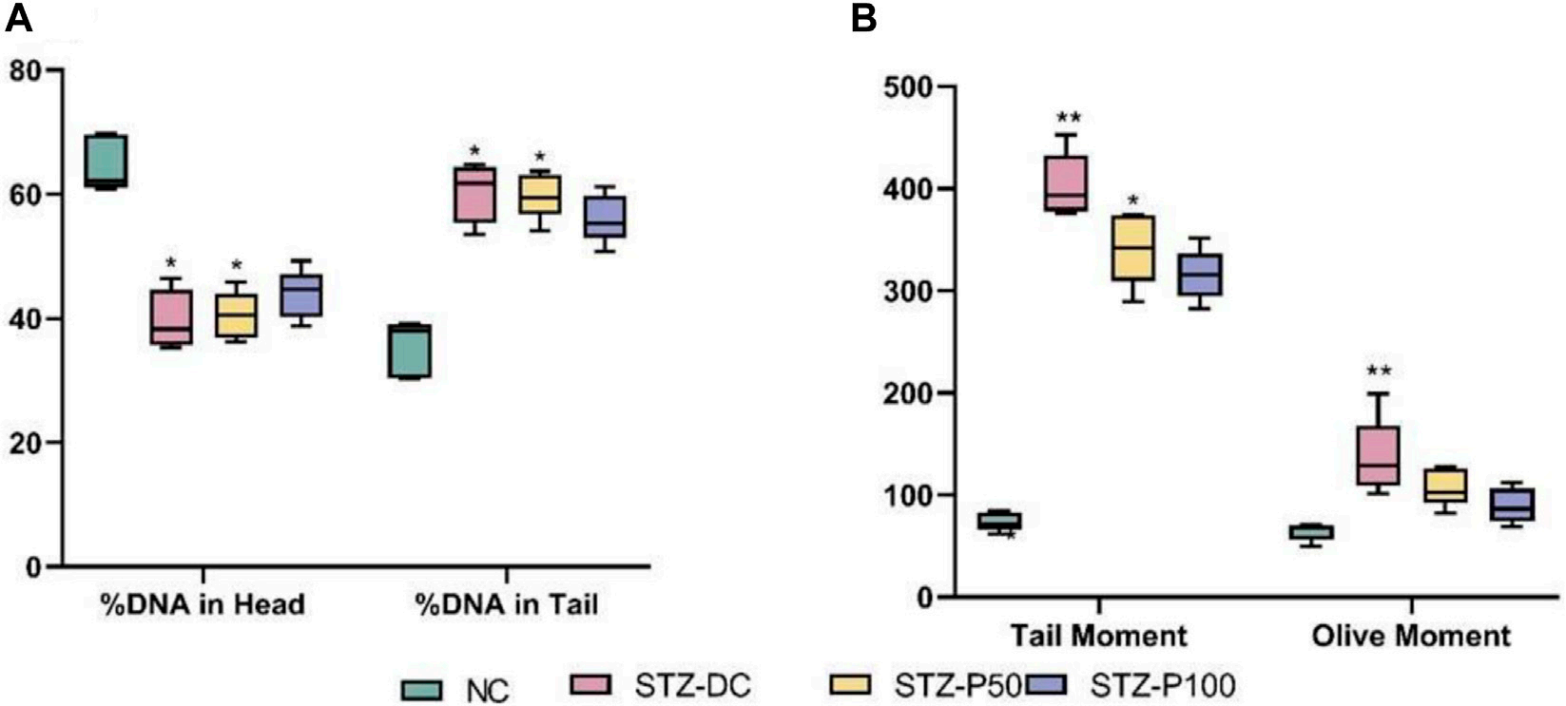
Effect of propolis on DNA integrity using comet assay. Data presented as median ±SEM (median of 5 samples/group; 50 cells/sample). ^*^Statistically significant from the NC group at *p* ≤ 0.05. Multiple group comparisons used Kruskal–Wallis’s test.

The results in Figure 12 clearly show that giving propolis to male diabetic rats prevented STZ-induced testicular DNA damage, particularly in the high dose group (STZ-P100). The DNA in both the head and tail was significantly decreased (Figure 12A); however, tail moment and olive moment were significantly increased in STZ-DC groups compared to the NC group. Propolis administration protected against DNA damage toward normal levels (Figure 12B).

## 4 Discussion

The present study was designed to determine the protective effects of propolis against testicular damage in diabetic rats induced by nicotinamide (NA) and streptozotocin (STZ). Additionally, rats given high-fat diets and low-dose streptozotocin are shown to have impaired insulin secretion, glucose intolerance, insulin resistance, and obesity, making them suitable as an alternate animal model for type 2 diabetes; hence, NA/STZ accompanied with HFD could serve as an appropriate animal model for type 2 diabetes (Tahara et al., 2011).

Type II diabetes was induced via the injection of NA/STZ in male rats fed with a high-fat diet for 8 weeks, resulting in a significant elevation of blood glucose levels by destroying the β-cells of Langerhans islets, lowering insulin levels, and raising HOMA-IR. Additionally, diabetic rats exhibited abnormalities in semen, with the increased production of reactive oxygen species (ROS) and DNA damage in testicular tissues. It is noteworthy that many previous studies support those findings.

Inducing type II diabetes in rats via NA/STZ injection is a well-established animal model, whereas STZ injection causes pancreatic B-cell damage, although NA partially protects insulin-secreting cells against STZ (Szkudelski, 2012; Abdel-Rahman et al., 2020a).

STZ, also referred to as a glucose analog, is a nitrosourea molecule. Due to its structural resemblance to glucose, STZ penetrates β cells through the glucose transporter-2 (Glut2), which is widely expressed on the surface of β cells. The nitrosoamide moiety of STZ is responsible for the genotoxicity and cytotoxicity of the compound once it enters β cells. It can target DNA and cause DNA alkylation. Therefore, the damage to DNA produced by STZ can activate PARP-1—poly (ADP-ribose) polymerase-1—which uses NAD + as a substrate to repair damaged DNA. Therefore, the activated PARP-1 may potentially deplete NAD+, which would result in cell death. NA administration prior to STZ could alleviate the deleterious effects of STZ via two methods: the direct inhibition of PARP-1 or acting as a precursor to NAD+ (Wu and Yan, 2015; Yan, 2022).

Propolis significantly decreased FBG levels compared to streptozotocin-diabetic control (STZ-DC) and significantly improved insulin levels. Consistent with our results, Samadi et al. and Zakerkish et al. verified that bee propolis causes a notable reduction in blood glucose, serum insulin, and glycosylated hemoglobin (HbA1c) levels, along with improved insulin resistance in type 2 diabetes patients (Samadi et al., 2017; Zakerkish et al., 2019). Additionally, Rifa’I verified that propolis increased insulin expression in pancreatic beta cells in STZ-induced diabetic mice (Rifa, 2017). Akdad et al. reported that the aqueous extract of Moroccan propolis displayed a strong antihyperglycemic effect in diabetic rats, attributable to its antioxidant capabilities (Akdad et al., 2021).

The STZ/DC group exhibited impaired spermatogenesis, as evidenced by notable reductions in sperm count, motility, and viability, as well as an increase in sperm with abnormal morphology (Table 2). The administration of propolis for 8 weeks at two dose levels (50 and 100 mg/kg) resulted in significantly increased sperm cell count, increasing the percentage of morphologically normal and viable sperm compared to STZ-DC group.

In support of these findings, male rat testicular damage was reduced and the detrimental effects of chlorpyrifos, an organo-phosphorous pesticide, were lessened by propolis administration prior to intoxication (ElMazoudy et al., 2011). Moreover, in Cedikova et al., propolis had the ability to enhance sperm motility by augmenting the overall mitochondrial respiratory efficiency of human spermatozoa *in vitro* (Cedikova et al., 2014). Furthermore, propolis can significantly improve the properties of rabbit sperm and protect against reproductive toxicity caused by the endocrine disruptor triphenyltin (Yousef et al., 2010).

In this study, the STZ-DC group revealed a significant decrease in serum total testosterone (TTST), estradiol (E_2_), follicle-stimulating hormone (FSH), luteinizing hormone (LH), and prolactin (PRL) compared with the NC group. Our data also revealed a substantial increase in the level of serum reproductive hormones (TTST, E_2_, FSH, LH, and PRL) in rats administered the high dose of propolis (100 mg/kg), as well as in testis homogenate (total testosterone, dihydrotestosterone, and estradiol) compared to STZ-DC rats.

One consequence of diabetes that affects male fertility is hypogonadism, which affects 20–64% of men with diabetes. Insulin, testosterone, and gonadotropins are all directly correlated. Inadequate insulin production has negative impact on the release of FSH and LH, while both gonadotropins stimulate the production of testosterone (Artimani et al., 2018).

Pituitary gonadotropin synthesis and secretion may be inhibited in diabetic rats due to hypothalamic and pituitary abnormalities, potentially leading to a drop in serum levels of LH and FSH (Sudha et al., 2000). Long-term type II diabetes mellitus (T2DM) in male patients has also been linked to decreased testosterone production, dysfunctions of the hypothalamic–pituitary–gonadal axis, and decreased fertility, as evidenced by studies on rodent models of these metabolic disorders and human patients (Bakhtyukov et al., 2021). According to Jackson and Hutson, decreased FSH and LH levels in serum imply that the hypothalamic–pituitary axis is adversely impacted by STZ-induced diabetes (Jackson and Hutson, 1984).

Male gonadal hormones, particularly LH and testosterone, are thought to be the main markers of susceptibility to exogenous reproductive toxicants and play a significant role in regulating spermatogenesis in male individuals (Banihani, 2023). LH, a peptide hormone secreted from the pituitary gland, stimulates Leydig cells, which then generate testosterone, a steroid hormone chemically derived from cholesterol. In the seminiferous tubules of the testis, it diffuses into Sertoli cells. Testosterone then attaches to androgen receptors in Sertoli cells’ cytoplasm and nucleus, inducing the physiological reactions required to promote spermatogenesis (Smith and Walker, 2014). Prolactin is a multipurpose compound that functions as both a cytokine and a circulating hormone. Prolactin receptor, as a member of the cytokine receptor superfamily, is involved in the activation of signal transduction that supports the development and survival of cells. Prolactin governs a variety of physiological activities through various pathways by influencing cellular processes like proliferation, differentiation, and cell survival. Additionally, prolactin may play a role in the feedback mechanism that notifies CNS centers that regulate sexual arousal and behavior, or it may be a peripheral regulatory component for male reproductive function (Esquifino et al., 2004).

Although PRL has demonstrated physiological levels for beta cell protection, low dosages of PRL improve the beta-cell function and insulin sensitivity of diabetic rats. Nonetheless, additional data suggest that metabolic syndrome, insulin resistance, and diabetic complications are linked to elevated PRL serum levels in T2DM patients (which is a risk factor for T2DM) (Duc Nguyen et al., 2022).

The results of the current study are corroborated by those of Nna et al., who found that Malaysian propolis enhances spermatogenesis, mating behavior, testicular lactate metabolism; steroidogenesis improves reproductive potential in diabetics (Nna et al., 2020).

Propolis has been observed to augment steroidogenesis in the testes of diabetic rats at the molecular level through up-regulating the mRNA and protein levels of cytochrome P450 A1 (CYP11A1), cytochrome P450 17A1 (CYP17A1), 3β-hydroxysteroid dehydrogenase (3β-HSD), and 17β-hydroxysteroid dehydrogenase (17β-HSD) (Xu et al., 2014; Nna et al., 2020).

In the present investigation, propolis supplementation (100 mg/kg) significantly improved the antioxidant profile in the rats’ serum and testicular homogenate, bringing it closer to normal values. In comparison to STZ-DC rats, there was a significant increment in the enzyme activities of CAT, SOD, and GPx and a significant decrease in MDA levels.

Remarkably, spermatozoa require a range of defensive mechanisms, including antioxidant enzymes such as glutathione (GSH) peroxidase and reductase, catalase (CAT), and superoxide dismutase (SOD) to balance the risks and benefits of ROS and antioxidants (Sikka et al., 1995).

It is widely recognized that anaerobic cells with a high concentration of mitochondria, such as spermatozoa, are susceptible to oxidative damage to their DNA. Additionally, phosphorylation and ATP synthesis are expected to be impacted by the redox state of human spermatozoa, which will have a significant impact on their capacity to fertilize. Accordingly, decreased sperm quality and male fertility are influenced by elevated ROS levels (Sikka et al., 1995; Cedikova et al., 2014).

Russo et al. proposed that propolis may be used to cure and prevent male infertility by defending human spermatozoa DNA from oxidative damage brought on by chemicals that react with thiobarbituric acid (TBARS) (Russo et al., 2006).

The strong antioxidant property in propolis has been found to be caused by a variety of bioactive constituents, especially phenolic ones such flavonoids (e.g., tectochrysin, chrysin, pinocembrin, galangin, apigenin, genkwanin, and kaempferol), stilbenes, flavan-3-ols (catechins), and hydroxybenzoic acids (Kurek-Górecka et al., 2013; Laaroussi et al., 2021).

In this study, the histopathologic investigation of testicular tissue of STZ-DC rats revealed a marked decrease in spermatogenic cells with vacuolation in Leydig cells. An excess of free radicals in diabetes may prevent Leydig cell androgen production and be the cause of abnormalities in testicular histology (Khaneshi et al., 2013). On the other hand, propolis-treated rats (STZ-P100) revealed a normal histology of seminiferous tubules and Leydig cells. The preventive action of propolis may be responsible for the decrease in testicular oxidative damage and the rise in serum reproductive hormones, particularly testosterone.

The immunohistochemical analysis of the testis of the STZ-DC group exhibited severe positive immune reaction against PCNA staining compared to the NC group, and negative expression for Bcl-2 in spermatogenic cells. Nevertheless, propolis at high dose level revealed negative expression for PCNA in spermatogenic cells and high positive reaction for Bcl-2 in spermatogenic cells (Figures 9, 10).

The immunoexpression of PCNA in the testis is considered a proliferative marker for estimating spermatogenesis. It has many values as a quick, reliable, sensitive, and quantitative method to determine and find early testicular toxicity. Findings of the current work confirmed those of previous reports of a significant increase of PCNA immunostaining in STZ-diabetic rats. The use of PCNA in evaluating germ cell kinetics and validating the histopathological results of the effects of diabetes on spermatogenesis was verified by Altay et al. (2003).

Furthermore, Bcl-2, a member of the Bcl-2 family of antiapoptotic proteins, plays a critical role in initiating the apoptosis pathway by preserving the integrity and functionality of the mitochondria. Previous studies have demonstrated that Bcl-2 is essential for normal spermatogenesis and controls whether damaged cells undergo apoptosis, corroborating our findings. Exposure of male rats to STZ leads to oxidative, cytotoxic, and genotoxic events along with the generation of ROS. ROS buildup and oxidizing enzyme activation damage the cell membrane and mitochondria and lead to germ cell death (Zhao et al., 2011; Zheng et al., 2023).

According to Rashid et al., bee propolis treatment reduced the expression of proliferation marker PCNA positive nuclei against diethylnitrosamine (DEN) initiated, and ferric nitrilotriacetate (Fe-NTA) promoted renal carcinogenesis in Wistar rats, which is consistent with the results of the current study (Rashid et al., 2013). These findings are also consistent with those of Sönmez et al. (2016), where propolis significantly increased PCNA positive cells in methotrexate-induced testis injury in rats. Moreover, propolis significantly increased Bcl-2 gene expression in doxorubicin-induced nephrotoxicity induced in male Albino rats (Mohamed et al., 2022).

**FIGURE 13:**
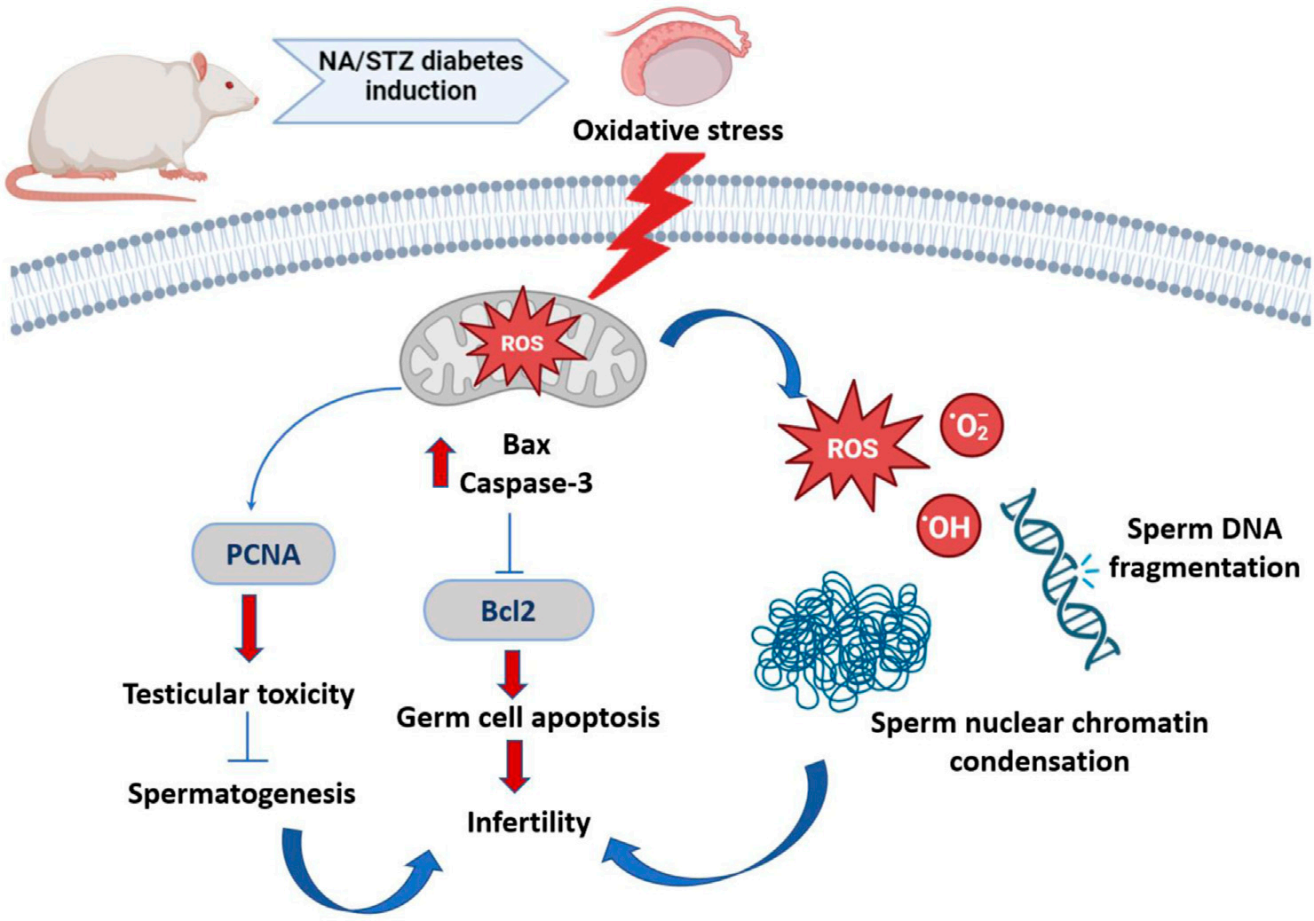
Schematic representation of diabetes-induced testicular oxidative stress. Diabetes inducing oxidative circumstances resulting in an increase in Bax and caspase 3 levels and inhibition of Bcl2 level, which subsequently induced apoptosis of germ cell via the Bax/Bcl2 and caspase pathway. Diabetes upregulates PCNA expression, resulting in testicular toxicity and suppressing spermatogenesis. Both quality and quantity of nuclear chromatin condensation and increase in sperm DNA fragmentation arise from diabetes, resulting in infertility.

Interestingly, both the quality and quantity of nuclear chromatin condensation and increase in sperm DNA fragmentation arise from diabetes (Figure 13) (Mangoli et al., 2013).

A notable improvement in DNA integrity was reported in this study, where propolis administration to rats prevented STZ-induced testicular DNA damage, particularly in the high dose group (STZ-P100), against normal levels.

Consistent with these findings, propolis protected genomic DNA from oxidative damage and decreased the extent of DNA damage induced by taxol, an anticancer agent that causes male reproductive toxicity (Abd-Elrazek et al., 2020).

The limitations to this study are that only two propolis dose levels were examined; further examination of more dose levels would be beneficial to detect the optimal dose. Additionally, further mechanistic investigation of the key regulators of the anti-oxidative response of propolis might be conducted.

## 5 Conclusion

Our findings indicate that propolis may have antioxidant capabilities that help mitigate testicular DNA damage and enhance sperm parameters (count, motility, morphology, and viability) in STZ-induced diabetic rats. Furthermore, it appears that the higher dose of propolis (100 mg/kg) is more effective in improving testicular damage and sperm parameters, thus recommending propolis as an adjuvant treatment option for male patients with diabetes.

## Data Availability

The datasets presented in this article are not readily available due to privacy and/or ethical restrictions. The datasets that support the findings of this study are available from the corresponding author upon reasonable request.
