# Supplementary material for: Propolis attenuates diabetes-induced testicular injury by protecting against DNA damage and suppressing cellular stress
Source: Front Pharmacol. 2024 Jul 11;15:1416238. doi: 10.3389/fphar.2024.1416238 (PMC11269134; doi:10.3389/fphar.2024.1416238)
Supplement: Supplementary file 1 [file DataSheet1.pdf]

Supplementary data

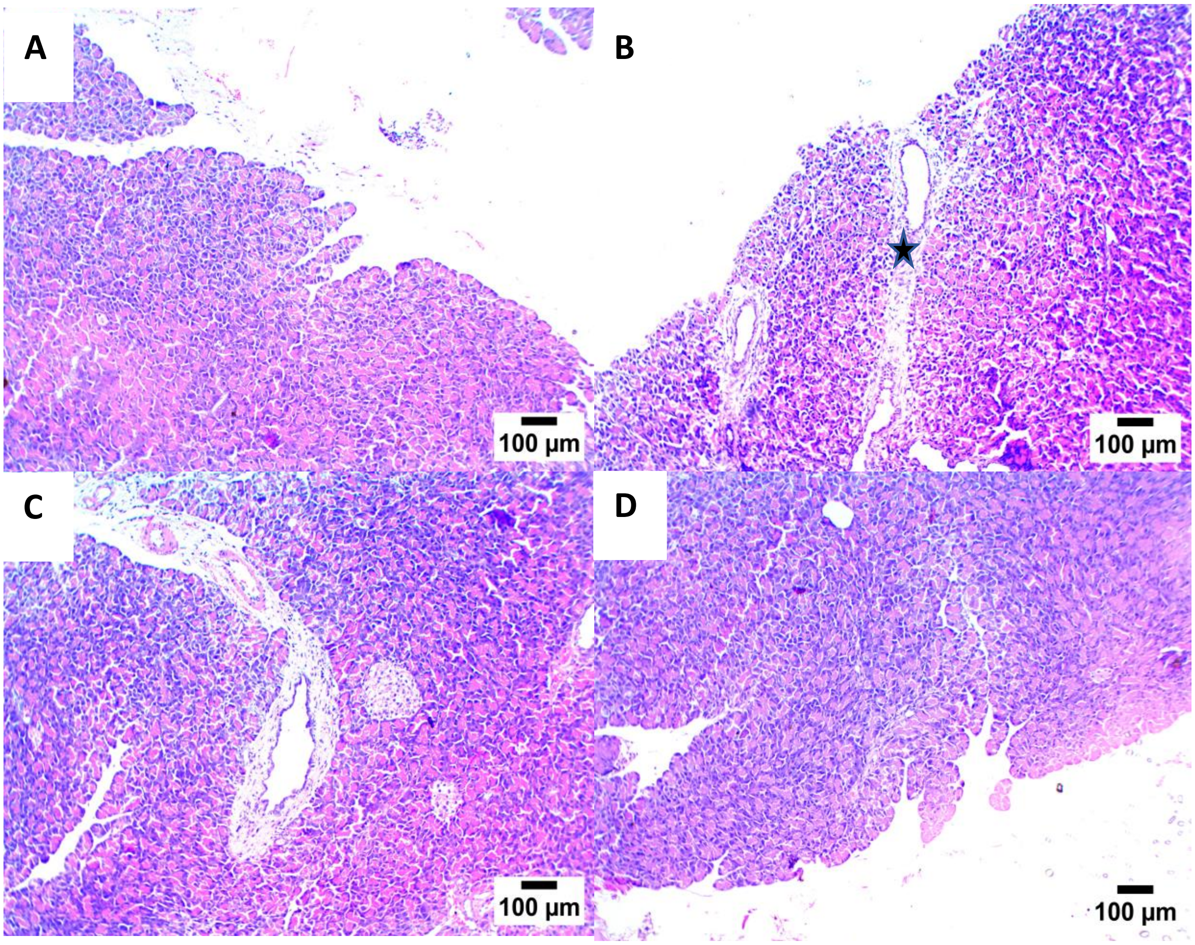

Figure (S1). photomicrograph showing A) section of NC group showing normal histological structure of pancreatic acini; B) section of STZ-DC showing preacinar fibrosis (star); C) section of STZ-P50 showing preacinar fibrosis (star); D) section of STZ-P100 showing mild preacinar fibrosis (arrow) (Hematoxylin and Eosin stain).

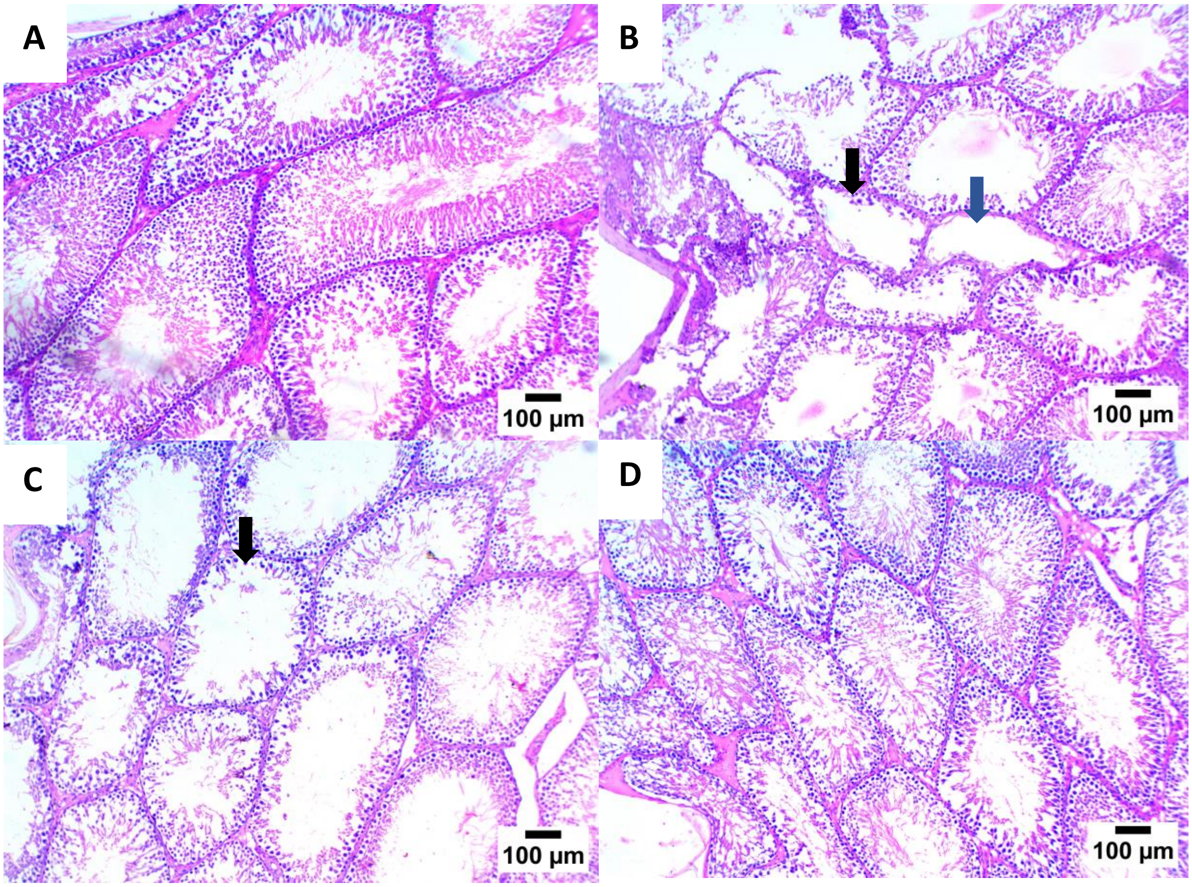

Figure (S2). photomicrograph showing A) section of NC group showing normal histological structure of seminiferous tubules; B) section of STZ-DC showing oligospermia in some seminiferous tubules (black arrow) and others showing azospermia (blue arrow); C) section of STZ-P50 showing oligospermia in some seminiferous tubules (arrow); D) section of STZ-P100 showing normal histological structure of seminiferous tubules.
